# Matrix Metalloproteinases and Arterial Hypertension: Role of Oxidative Stress and Nitric Oxide in Vascular Functional and Structural Alterations

**DOI:** 10.3390/biom11040585

**Published:** 2021-04-16

**Authors:** Alejandro F. Prado, Rose I. M. Batista, Jose E. Tanus-Santos, Raquel F. Gerlach

**Affiliations:** 1 Laboratory of Structural Biology, Institute of Biological Sciences, Federal University of Para, Belem, PA 66075-110, Brazil; alejandrofp@ufpa.br; 2 Department of Pharmacology, Ribeirao Preto Medical School, University of Sao Paulo, Ribeirao Preto, SP 14049-900, Brazil; rosebatista19@usp.br (R.I.M.B.); tanus@fmrp.usp.br (J.E.T.-S.); 3 Department of Morphology, Physiology and Basic Pathology, Faculty of Dentistry of Ribeirao Preto, University of Sao Paulo, Ribeirao Preto, SP 14040-904, Brazil

**Keywords:** EGFR, NADPH oxidase, vasoconstriction, endothelial dysfunction, phenylephrine, angiotensin II, vascular remodeling, fibrosis

## Abstract

Various pathophysiological mechanisms have been implicated in hypertension, but those resulting in vascular dysfunction and remodeling are critical and may help to identify critical pharmacological targets. This mini-review article focuses on central mechanisms contributing to the vascular dysfunction and remodeling of hypertension, increased oxidative stress and impaired nitric oxide (NO) bioavailability, which enhance vascular matrix metalloproteinase (MMP) activity. The relationship between NO, MMP and oxidative stress culminating in the vascular alterations of hypertension is examined. While the alterations of hypertension are not fully attributable to these pathophysiological mechanisms, there is strong evidence that such mechanisms play critical roles in increasing vascular MMP expression and activity, thus resulting in abnormal degradation of extracellular matrix components, receptors, peptides, and intracellular proteins involved in the regulation of vascular function and structure. Imbalanced vascular MMP activity promotes vasoconstriction and impairs vasodilation, stimulating vascular smooth muscle cells (VSMC) to switch from contractile to synthetic phenotypes, thus facilitating cell growth or migration, which is associated with the deposition of extracellular matrix components. Finally, the protective effects of MMP inhibitors, antioxidants and drugs that enhance vascular NO activity are briefly discussed. Newly emerging therapies that address these essential mechanisms may offer significant advantages to prevent vascular remodeling in hypertensive patients.

## 1. Hypertension Is a Common Disease Leading to a Variety of Cardiovascular Complications and Presenting a Significant Public Health Problem

Cardiovascular diseases (CVDs) are the greatest causes of death globally. More people die from CVDs annually than any other cause [1]. CVDs include coronary heart disease, heart failure, stroke and hypertension. In 2017, 17.8 million deaths were attributed to CVDs globally, an estimated 31% of all deaths worldwide. Most CVD deaths are due to heart attacks or strokes [1]. High blood pressure, which is characterized by systolic blood pressure ≥140 mm Hg and diastolic blood pressure ≥90 mm Hg [2], is a significant risk factor for heart attacks and CVDs [1]. Therefore, hypertension is still a leading cause of death globally, accounting for 10.4 million deaths in 2017 [3].

## 2. Basic Pathophysiological Mechanisms Contributing to the Vascular Alterations of Hypertension

Hypertension is a complex multifactorial disorder involving pathophysiological mechanisms that interact with each other to promote functional and structural modifications of the cardiovascular system. Sustained high blood pressure results from the combination of various genetic and environmental alterations that have neuroendocrine, hemodynamic, inflammatory and tissue redox components. This mini-review article will focus on critical mechanisms contributing to the vascular remodeling of hypertension: increased oxidative stress both impairing NO bioavailability and enhancing vascular MMP activity.

Oxidative stress results from the overproduction or inhibited inactivation of reactive oxygen species (ROS), causing alterations in the redox state of proteins and other mediators [4]. The most critical ROS in the cardiovascular system are superoxide anions (O_2_^−^), hydrogen peroxide (H_2_O_2_), nitric oxide (NO) and peroxynitrite (ONOO^−^) [5]. Increased ROS formation triggers protein oxidation and activation of a cascade of cell signaling events, resulting in endothelial dysfunction and MMP activation [6,7,8]. In fact, there is strong evidence that the biochemical alterations mentioned here underlie critical mechanisms leading to endothelial dysfunction and vascular remodeling in hypertension. 

MMPs are calcium and zinc-dependent enzymes that play a crucial role in the degradation of extracellular matrix components [9,10], membrane proteins [11,12,13], peptides [14,15] and intracellular proteins [16,17,18,19,20,21,22]. Proteolytic MMP activity increases blood pressure, causes vasoconstriction and endothelial dysfunction and promotes cell migration with vascular remodeling in hypertension [12,17,18,19,23,24,25,26,27,28,29,30,31]. In fact, it is now widely acknowledged that the interaction between ROS and MMPs tends to increase vasoconstriction and attenuate endothelial relaxation mediated by vasoactive agents [12,32,33]. Phenylephrine (PE), angiotensin II (Ang-II), and endothelin-1 (ET-1) promote vasoconstriction at least in part by inducing ROS production, and this mechanism is essential to maintain vasoconstriction [4,12,34,35,36,37,38]. Another important mechanism associated with increased oxidative stress is the reduced bioavailability of an endothelium-derived relaxing factor (EDRF) such as NO, which leads to endothelial dysfunction. This may result from NO rapidly reacting with several other ROS, particularly O_2_^−^, forming ONOO^−^ [5], which enhances protein nitration and contributes to many pathophysiological mechanisms taking place in the vasculature of hypertensive subjects [4]. NO is a vasodilator produced by three synthases: endothelial (eNOS), neuronal (nNOS) and inducible (iNOS). These synthases convert L-arginine and molecular oxygen to produce NO and L-citrulline using the cofactors tetrahydrobiopterin (BH4), flavin adenine dinucleotide (FAD), flavin mononucleotide (FMN) and nicotinamide adenine dinucleotide phosphate (NAD) [39]. Impaired NO availability in hypertension is also due to increased concentrations of asymmetric dimethylarginine, which competes with L-arginine for endothelial NO synthase, inhibiting NO formation [40,41], or endothelial NO synthase uncoupling, thus making this enzyme another source of O_2_^−^ instead of NO [42]. In contrast, the increase in iNOS expression has detrimental effects on hypertension, as the excess NO produced by this isoform reacts with O_2_^−^, forming ONOO^−^, and leads to oxidative damage. iNOS can also regulate arginase activity, reducing NO bioavailability by uncoupling eNOS, with enhanced production of O_2_^−^ instead of NO [43]. Increased iNOS expression was found in aortas and hearts from rats [44] and from patients with heart failure [45,46]. There is also increased nitrotyrosine (a marker of ONOO^−^ formation) in the aortas and hearts of hypertensive animals. Moreover, iNOS knockout mice submitted to myocardial infarction show lower mortality and less oxidative stress and cardiac remodeling when compared to wild-type mice [47,48].

These critical mechanisms involving increased MMP activity and tissue ROS concentrations, associated with impaired NO activity, interact with other cellular signaling pathways that cause both functional and proliferative alterations in the vasculature of hypertensive subjects.

## 3. An Overview of MMPs’ Regulation by Oxidative Stress and NO Bioavailability

MMPs are initially synthesized in a latent pro-form as zymogens. The basic structure of the catalytic portion of proteases consists of a catalytic domain with three histidine residues linked to a zinc atom (Zn^2+^) and a pro-domain containing a cysteine. In the inactive state, the cysteine residue interacts with the zinc ion, maintaining MMP latency [49]. Enzymatic activation is required to cleave the pro-domain and to expose the catalytic site [50]. MMP activation may also occur as a result of the disruption of zinc interaction with cysteine in the pro-domain [51,52,53], arising from enhanced ROS formation, for example [6,7,8]. 

**Figure 1:**
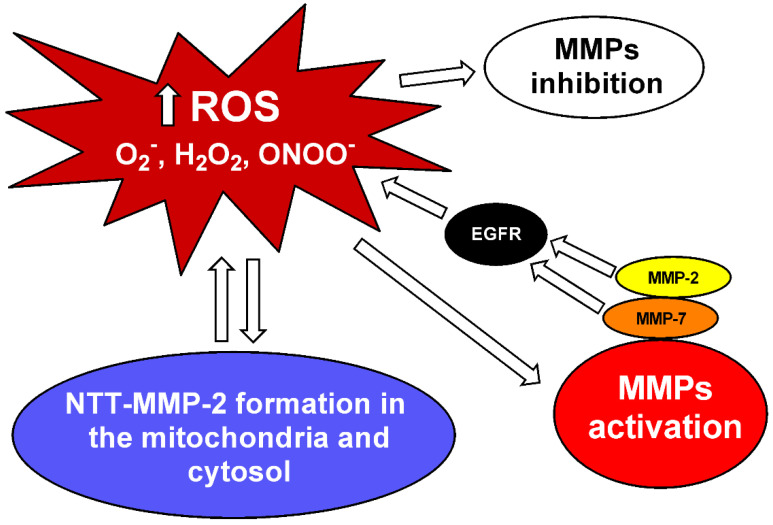
MMP inhibition and interaction between MMPs and reactive oxygen species (ROS). Increased levels of superoxide (O_2_^−^), hydrogen peroxide (H_2_O_2_) and peroxynitrite (ONOO^−^) can activate matrix metalloproteinases (MMPs) [6,7,8]. MMP-2 and MMP-7 activate pro-oxidant pathways in the vascular tissue via epidermal growth factor receptor (EGFR) transactivation [12,69]. ROS can also lead to the formation of a truncated MMP-2 (NTT-MMP-2) with 65 kDa located in the mitochondria and cytosol, which downregulates genes associated with resistance to oxidative stress [67]. High ROS concentrations may decrease MMP activity [7,8].

It has been shown that changes in the tissue concentrations of O_2_^−^, H_2_O_2_ and ONOO^−^ affect MMP-2 activity [7,8]. High concentrations of H_2_O_2_ and ONOO^−^ decrease MMP-2 activity, whereas low concentrations of these ROS increase MMP-2 activity [7,8] (Figure 1). The antioxidant activity of the enzyme catalase protects against the loss of MMP-2 activity, induced by high concentrations of H_2_O_2_ [7]. Likewise, in the presence of glutathione, the increase in MMP-2 activity induced by ONOO^−^ does not occur [8]. In this respect, ONOO^−^ has been shown to activate the MMP-2 zymogen in a concentration-dependent manner, with low concentrations (<1 µM) increasing and high concentrations (3–100 µM) decreasing MMP-2 activity. This effect involves the ONOO^−^ mediated S-glutathionylation of the critical cysteine residue of 72 kDa MMP-2 [8]. Interestingly, more complex interactions have been shown between ONOO^−^ and glutathione, affecting MMP-2 phosphorylation, which is another post-translational modification of MMP-2, mediated by protein kinase C (PKC), which may affect MMP-2 activity [54,55]. The decrease in MMP-2 activity can result in physiological changes. Patients with MMP-2 deficiencies have bone, joint and congenital heart problems [56,57,58,59]. In some clinical situations with increased oxidative stress, such as sepsis, decreased MMP-2 activity has been shown in the heart perfusate and the left ventricles of rats after lipopolysaccharide (LPS) infusion [60]. It is known that MMP-2 plays a role in the dysfunction found in these organs. However, its role has yet to be further elucidated. Moreover, MMP-2 knockout mice show a pro-inflammatory phenotype [61,62,63]. On the other hand, increased MMP-2 activity also increases inflammation. These previous findings should be taken into consideration when testing MMP inhibitors, as excessive MMP inhibition may also promote inflammation. For a more in-depth review of this issue, please read [64].

The activation of MMP-2 by ONOO^−^ leads to the formation of active 72 kDa intracellular MMP-2 [8], which is implicated in the degradation of critical intracellular targets in the contractile machinery of cardiomyocytes during ischemia and reperfusion, such as troponin I [22], alpha-actinin [21], titin [16] and light chain myosin [20], probably leading to heart failure. This is very important to the cardiovascular system as a whole, as the deleterious consequences of MMP-2 activation are not limited to the heart [65]. In fact, MMP-2 activation by oxidative stress has been shown in the aortas of two-kidney, one-clip (2K-1C) hypertensive rats in association with decreased calponin-1 expression, resulting in abnormal vascular smooth muscle cells’ (VSMCs’) proliferation and hypertrophic remodeling [17,18,19]. However, treatment with nonspecific inhibitor doxycycline, dose-dependently [66], prevented MMP-2 increased activity and calponin loss [17,19]. The antioxidant tempol, which previously was shown to decrease vascular MMP-2 activity in hypertension [28], prevented calponin-1 loss in 2K-1C hypertensive rats, possibly by decreasing MMP-2 S-glutathionylation [18]. Further supporting the relationship between an imbalanced redox state and abnormal MMP-2 activity, increased ROS concentrations have been shown to promote the formation of a truncated MMP-2 isoform (NTT-MMP-2) with 65 kDa located in the mitochondria and cytosol. Lovett et al. showed that NTT-MMP-2 overexpression in a cardiomyocyte downregulated the B-cell lymphoma-extra-large (BcL-xL) and heat shock protein family D1 (HSPD1), which are genes associated with resistance to oxidative stress [67] (Figure 1). Moreover, transgenic MMP-2 expression in mice shows increased lipid peroxidation in the heart after ischemia-reperfusion [68].

More recently, our group showed evidence supporting the idea that increased MMP-2 activity in VSMC directly activates pro-oxidant pathways. MMP-2 cleaves pro-heparin binding epidermal growth factor (HB-EGF) and soluble HB-EGF, in turn, transactivates the EGF receptor (EGFR). This kinase receptor activates phospholipase, PKC and NADPH oxidase, which increases ROS formation [12] (Figure 1). Barhoumi et al., using MMP-2 knockout mice, showed that MMP-2 is required for Ang-II to increase perivascular ROS formation in an animal model with the infusion of Ang-II (Figure 1). Interestingly, MMP-2 knockout impairs the vascular dysfunction and oxidative stress induced by Ang-II without affecting the increases in blood pressure induced by this mediator [32]. In addition to MMP-2, MMP-7 also activates pro-oxidant pathways in vascular tissue via EGFR transactivation [69] (Figure 1).

While the relationship between increased ROS concentrations and MMP activation is relatively clear, the same is not true with NO. Besides its vasodilatory effects, NO also inhibits platelet aggregation, VSMC migration and proliferation and is involved in the regulation of extracellular matrix composition [70]. The direct relation between NO and MMPs, especially MMP-9, seems to be conflicting, indicating a dual role of the molecule in MMP-9 regulation [71]. While NO can activate MMP-9 in rat retinal neurons, the NO donor spermine-NONOate decreases MMP-9 activity in endothelial and carcinoma co-cultures [72,73]. It is possible that this apparent discrepancy is explained by differences (low versus high) in NO concentrations [71]. There is clinical evidence supporting the notion that endogenous NO production, assessed by circulating nitrite concentrations, is inversely related to the circulating concentrations of MMP-2 and MMP-9 [74,75].

Overall, the studies discussed above support the idea that increased vascular oxidative stress has a direct relationship with imbalanced MMP activity, and this mechanism may involve impaired NO bioavailability. Together, these mechanisms may underlie both functional and structural alterations commonly found in the vessels of hypertensive subjects. 

## 4. Evidence Indicating That Increased Vascular MMP Activity Promotes Vascular Dysfunction in Hypertension

Increased vascular MMP-2 expression and activity has been shown in a variety of hypertensive animal models, both in conductance and resistance arteries [27,76,77]. This biochemical alteration is associated with increased responsiveness to vasoconstrictors, such as PE, and reduced responsiveness to vasodilators, such as acetylcholine, both prevented by MMP inhibitors [19,27]. Confirming these findings, endothelial dysfunction is observed in wild-type mice after Ang-II treatment but not in MMP-2 knockout mice [32]. Given that antioxidant drugs and MMP inhibitors reverse these functional vascular alterations associated with increased vascular MMP-2 activity, it is now evident that increased ROS formation plays an important role in promoting these pathophysiological mechanisms in the vessels from hypertensive animals [12,26,28]. 

**Figure 2:**
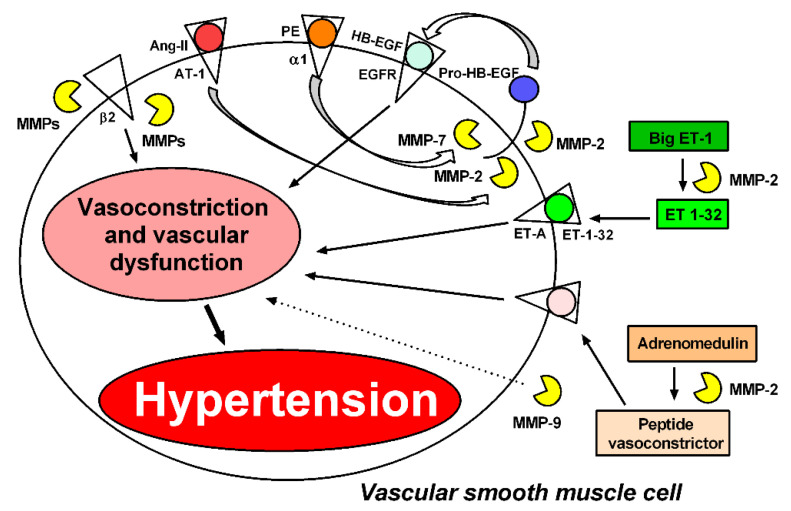
MMPs induce vasoconstriction and vascular dysfunction through different mechanisms. Phenylephrine (PE), angiotensin II (Ang-II), and endothelin-1 (ET-1) mediate vasoconstriction by generating reactive oxygen species (ROS); this mechanism is essential to maintain vasoconstriction. MMP-2 and MMP-7 are required for Ang-II and PE to induce vasoconstriction by transactivation of the epidermal growth factor receptor (EGFR) [12,69]. MMP-2 also induces vasoconstriction by cleaving big endothelin-1 (Big ET-1) to endothelin 1–32 (ET 1–32) [14]. The action of MMP-2 on adrenomedullin leads to the formation of metabolites with vasoconstrictive activity [78]. MMPs (not identified yet) cleave the beta-2 adrenergic receptor, contributing to increased arteriolar tone in spontaneously hypertensive rats [30]. MMP-9 can increase the vasoconstriction of arterioles and venules by a mechanism not yet clarified [30]. These processes may contribute to hypertension.

As discussed above, we have previously shown that MMP-2 cleavage of pro-HB-EGF results in the transactivation of the HB-EGF receptor and intracellular signaling, increasing ROS formation [12]. This mechanism may explain how increased MMP-2 activity increases the vascular reactivity to PE or other vasoconstrictors, including Ang-II, as previously shown [32], possibly by directly stimulating ROS formation [36] (Figure 2). Interestingly, while ROS formation is usually accepted as an upstream mechanism of MMP activation, there is now growing evidence indicating that MMP activation may be upstream of ROS production [12,33,68,69].

**Figure 3:**
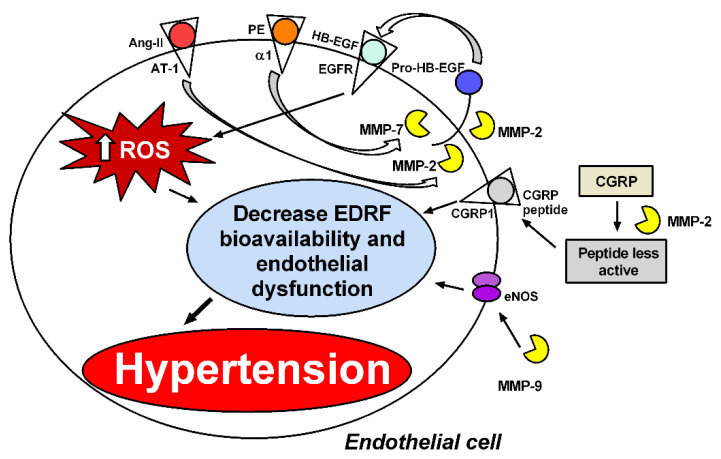
MMPs induce endothelial dysfunction by different mechanisms. MMP-2 and MMP-7 are required for phenylephrine (PE) to induce reactive oxygen species (ROS) by transactivation of the epidermal growth factor receptor (EGFR) [12,69]. MMP-2 is also required for angiotensin II (Ang-II) to increase perivascular ROS formation [32]. MMP-2 cleaves the vasodilator peptide related to the calcitonin gene (CGRP) into peptides with more minor vasodilatory effects, and MMP-9 decreases endothelial nitric oxide synthase (eNOS) expression and vasodilatation in resistance arteries [80]. ROS decrease endothelium-derived relaxing factors’ (EDRFs’) bioavailability, such as NO, leading to endothelial dysfunction and hypertension.

Increased ROS activity reduces NO bioavailability, particularly in hypertension [79], by mechanisms discussed above. The improvement in the endothelium-dependent vascular responses to acetylcholine reported in hypertensive rats treated with doxycycline may reflect improved NO activity resulting from lower NO inactivation by ROS when those animals are treated with this MMP inhibitor [26]. While most studies have implicated MMP-2 as a major cause of vascular dysfunction in hypertension, MMP-9 is apparently also involved. In fact, increased endothelium-dependent vasodilation is described in resistance arteries from MMP-9 knockout mice, and this finding is associated with increased expression of endothelial NO endothelial synthase [80]. In addition to causing increased ROS formation and reduced NO activity, imbalanced vascular MMP activity promotes vascular dysfunction by activating other mechanisms (Figure 3).

MMP-mediated proteolytic activity degrades peptides and proteins (particularly receptors) that are involved in the control of vascular tone and blood pressure regulation, and, therefore, may promote hypertension. Rodrigues et al. have shown that MMPs cleave the beta-2 adrenergic receptor, contributing to increased arteriolar tone in spontaneously hypertensive rats (SHR) [30]. Interestingly, MMP-2 infusion also impairs responses to beta-1 adrenergic receptor stimulation [81]. However, the involvement of MMPs in blood pressure regulation remains unclear. Treatment with the nuclear factor kappaB (NF-kB) inhibitor pyrrolidine dithiocarbamate (PDTC) blocks this cleavage process, suggesting that NF-kB increases the activity of MMPs [82], resulting in cleavage of the beta-2 adrenergic receptor [31] (Figure 2).

MMP-2 and MMP-7 are the most widely studied MMPs involved in functional alterations of the cardiovascular system. Previous studies have shown that MMP-2 contributes to impairing blood pressure regulation by cleaving big endothelin-1 (Big ET-1) and generating a potent vasoconstrictor, endothelin 1–32 (ET 1–32) [14]. Moreover, MMP-2 degrades adrenomedullin, leading to the formation of less potent vasodilatory metabolites and some vasoconstrictors [78] (Figure 2). It has also been demonstrated in vitro that MMP-2 cleaves the vasodilator peptide related to the calcitonin gene (CGRP) into peptides with fewer vasodilatory effects [15] (Figure 3). In addition to MMP-2-mediated vasoconstriction and impaired vasodilation, MMP-7 has been shown to promote the vasoconstriction of resistance arteries. Hao et al. have shown that MMP-7 stimulates the vasoconstriction of mesenteric arteries by mechanisms that are dependent on EGFR transactivation, which are essential for maintaining vascular tone [69] (Figure 2). Further supporting this idea, in vivo injection of MMP-7 and MMP-9, individually or in combination, into microvessels of Wistar rats causes the rapid vasoconstriction of venules and arterioles [30] (Figure 2).

The studies discussed above show strong evidence that hypertension is associated with increased vascular MMP activity, resulting in impaired vascular function by a variety of mechanisms affecting vascular biology. Chronic hypertension, however, is associated with several structural alterations to the vasculature. 

## 5. Structural Alterations in the Vasculature Associated with Increased MMP Activity

Structural modifications usually found in the arteries of hypertensive animals are considered adaptive responses to changes in hemodynamic or metabolic demands. However, the progression of such modifications causes maladaptive alterations that can culminate in the pathological vascular alterations observed in cardiovascular diseases [83]. In hypertension, the rearrangement of vascular wall components is caused by changes in blood-pressure-induced circumferential wall stress and blood-flow-induced shear stress, which lead to modifications of the vascular extracellular matrix composition and changes in the cellular secretion of endogenous cytokines, with enhanced sensitivity to circulating humoral factors [25,84]. 

Several studies correlate circulating MMP concentrations and hypertension [85,86,87,88]. The activation of MMPs, especially MMP-2 and MMP-9, is directly involved in the vascular remodeling observed in hypertension since they are responsible for the degradation of extracellular matrix proteins, including elastin and collagen, and promote migration and phenotypic alterations of VSMC in resistance and conductance arteries [84]. These modifications result in the accumulation of collagen degradation products and increase vascular wall stiffness [84]. Vascular remodeling is a hallmark of a vascular disease’s severity and progression and is critically associated with relevant clinical events and the prognosis [89].

## 6. Imbalanced Vascular MMP Activity Activates Critical Mechanisms Leading to Vascular Remodeling in Hypertension

While vascular hypertrophy, usually found in the vessels of hypertensive subjects, tends to normalize vascular wall tension, it involves major changes in the endothelial and VSMC, as well as in the composition of the extracellular matrix [90]. In essential hypertension, eutrophic remodeling occurs in small arteries (diameter < 300 μm), which is associated with a reduced lumen and external diameter, increasing the media thickness to lumen diameter (M/L) ratio without significant changes to the total amount of wall tissue (whole cross-sectional area is maintained) or vessel wall material [91]. Vascular remodeling of these arteries prevents an increase in wall stress at the level of arterioles and capillaries [92]. In conductance arteries, however, hypertrophic remodeling results in augmented M/L ratios and cross-sectional areas, which are associated with VSMC growth (increased volume) or hyperplasia (increased cell number), collagen deposition and elastin degradation. These alterations cause increased vessel stiffness and decreased arterial compliance, negatively impacting the myocardial work capacity and coronary perfusion [93].

In addition to the functional alterations promoted by MMPs in the vasculature that are discussed above, these proteases also play critical roles in the vascular remodeling elicited by stimuli such as oxidative stress, inflammation, Ang-II and hemodynamic forces in hypertension [94]. In fact, mechanical forces and vasoconstrictor agonists have synergistic effects on the expression and activity of MMPs in hypertension [95]. Increases in both the MMP-2 and MMP-9 concentrations and activity assessed in the vessels or in the plasma of hypertensive animals or humans are apparently not accompanied by corresponding increases in the activity of endogenous MMP inhibitors (the tissue inhibitors of matrix metalloproteinases), thus revealing imbalanced MMP activity in hypertension [77,85,86,88]. In addition to increasing the degradation of the extracellular matrix components, these alterations stimulate vascular smooth muscle cells to switch from contractile to synthetic phenotypes, thus facilitating cell growth or migration [96]. This phenotype switch, which is associated with variable deposition of extracellular matrix components (collagen, proteoglycan, fibronectin and elastin) [90] is prevented by MMP inhibitors [27,93].

Many bioactive molecules—such as Ang-II, ET-1, aldosterone and catecholamines—impact MMP activity and vascular remodeling, at least in part, because of their capacity to increase oxidative stress and to impair NO activity in the vascular wall [97]. Ang-II activates vascular NADPH oxidase in the VSMC, increasing O_2_^−^ formation [98], and activates vascular MMP, as discussed above. ET-1 has been shown to enhance MMP-2 activity in mesangial cells, and the blockade of ET-1 type A receptors reduces MMP-2 and MMP-9 in the hearts of deoxycorticosterone acetate (DOCA)-salt hypertensive rats [99,100]. Although aldosterone apparently does not directly activate MMPs, it has been shown that eplerenone, a selective mineralocorticoid receptor antagonist, reduces ventricular MMP-2 and MMP-9 activities in dogs [101,102]. Moreover, the non-selective aldosterone antagonist, spironolactone, reduces vascular MMP-2 activity in hypertensive rats [103]. Catecholamine norepinephrine increases cardiac MMP-2 activity and collagen deposition [104], thus indicating that adrenergic receptors’ activation enhances MMP activity. In fact, the antagonist of β-adrenergic receptors, nebivolol, attenuates hypertension-induced vascular remodeling and increases vascular MMP-2 activity [105]. Similar effects are also reported in the hearts of hypertensive animals [106]. 

MMP-2 and MMP-9 are also responsible for activating cytokines, such as transforming growth factor-β (TGF-β), which are involved in collagen accumulation and profibrotic alterations in remodeling that are associated with hypertension [105]. Tumor necrosis factor-α (TNF-α) is another cytokine implicated in the vascular remodeling of hypertension, as it is essential for Ang-II-induced MMP-2 expression [107]. Finally, another mechanism that contributes to the VSMC phenotype switch in hypertension is the MMP-2-induced degradation of calponin-1, as recently shown [17,19].

MMP-1, MMP-2, MMP-3 and MMP-9 are the MMPs most frequently found at increased concentrations in hypertensive patients [68,108,109,110,111]. In fact, increased plasma levels of MMP-9 correlate with increased cardiovascular risk, myocardial infarct mortality, cardiac remodeling and dysfunction [112,113,114,115,116]. MMP-2 is also associated with cardiac failure, and both genetic polymorphisms commonly found in the MMP-2 gene [117,118,119,120,121,122] and increased plasma MMP-2 concentrations [123,124,125,126,127,128] are linked to heart failure. On the other hand, MMP-9 and its endogenous inhibitor, tissue inhibitor of metalloproteinase (TIMP)-1, when measured in serum, have been shown at increased concentrations. The MMP-9/TIMP-1 ratio (an index of net MMP-9 activity) is associated with great arterial stiffness, as assessed by carotid-femoral pulse wave velocity, but not with muscular arterial elasticity, as assessed by carotid-radial pulse wave velocity (CRPWV), in patients with hypertension [110]. Yasmin et al. showed increased MMP-2, MMP-9 and serum elastase activity in hypertensive patients. These findings were associated with aortic stiffness; the higher MMP-9 levels were an independent predictor of aortic stiffness [111]. Zhou et al. showed that polymorphism of MMP-9 in never-treated hypertensive patients was associated with increased blood pressure and aortic stiffness [129]. In addition, Stakos et al. showed that aortic stiffness in patients with arterial hypertension was associated with higher proMMP-1 and a higher proMMP-1/TIMP-1 ratio [109]. Together, these studies clearly indicate the involvement of various MMPs in hypertension.

## 7. MMP Inhibition as a Therapeutic Strategy to Prevent Vascular Remodeling in Hypertension

The huge number of studies indicating that vascular remodeling of hypertension is associated with abnormal vascular MMP activity, matrix deposition and oxidative stress and decreased NO activity suggest that these critical mechanisms are important pharmacological targets to explore in order to prevent or to treat the vascular alterations of hypertension. In the sections above, we discussed a variety of studies using MMP inhibitors or antioxidant drugs that may blunt MMP activation. 

**Figure 4:**
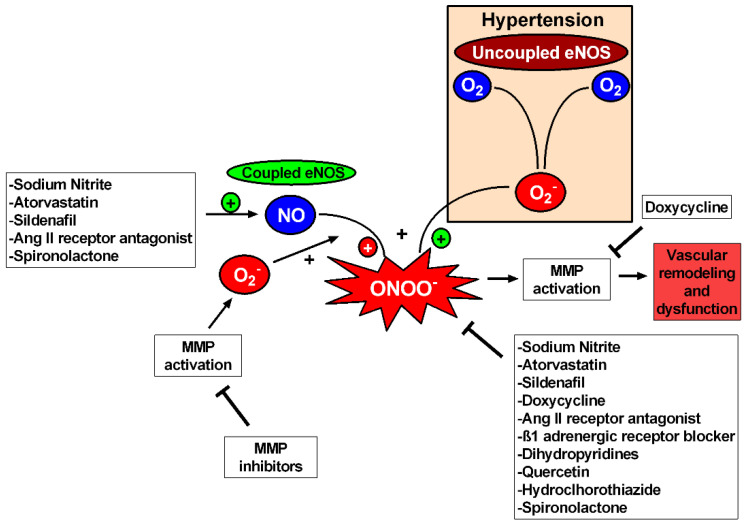
Oxidative stress inhibition reduces MMP activation in hypertension. Uncoupled eNOS and MMPs produce superoxide (O_2_^−^) that uses NO for peroxynitrite (ONOO^−^) production causing MMP activation and consequently vascular remodeling and dysfunction. Thus, MMP inhibitors and drugs with antioxidant properties can inhibit MMP activation, improving vascular function and reverting vascular remodeling. Doxycycline has antioxidant effects but also relies on direct inhibition of the active forms of MMPs. Drugs capable of augmenting NO bioavailability are also related to MMP inhibition, but further studies are needed to establish the relation between augmentation in NO (and NO-related species) and MMPs in hypertension.

While MMP inhibitors or antioxidants directly interfere with vascular MMP activation, many other studies show that other commonly used drugs may also attenuate vascular MMP activation and prevent the vascular remodeling of hypertension by exerting pleiotropic effects not directly associated with their canonical mechanism of action (Figure 4). For example, the antagonist of β1-adrenergic receptors, nebivolol, decreases vascular MMP-2 activity and attenuates hypertension-induced vascular remodeling and collagen deposition by mechanisms apparently independent of β1 adrenoceptor-blocking properties, probably related to diminished NADPH oxidase activity decreasing ROS formation [103,106]. Additional examples of drugs exerting such beneficial pleiotropic effects include some dihydropyridines—such as nifedipine, nimodipine and amlodipine—which have antioxidant effects and downregulate MMP [130,131,132], or losartan, an antagonist of Ang-II type 1 receptors [133]. Other drugs with antioxidant effects, such as quercetin, also exert anti-MMP and anti-remodeling effects in hypertension [29]. 

Finally, recent studies have shown that therapeutic strategies to increase NO activity include statins or the phosphodiesterase-5 inhibitor, sildenafil [134]. Inorganic nitrite [135,136] or nitrate [96] supplementation may also exert antioxidant effects and diminish MMP-2 activity and the vascular remodeling of hypertension. Importantly, oral nitrite treatment prevents hypertension-induced vascular oxidative stress and remodeling independently of its anti-hypertensive effects [135], thus suggesting that these beneficial effects are not dependent on blood pressure. Together, these studies strongly suggest a relationship between increased NO availability, decreased oxidative stress, attenuated vascular MMP activity and protection against vascular remodeling in hypertension.

First-generation MMP inhibitors are nonspecific. They act by binding to zinc in the catalytic domain present in all MMPs, thus decreasing its selectivity. These compounds have chemical instability bonding [137,138]. The low selectivity of these compounds is attributable to the fact that zinc is present in other proteases, such as disintegrin and metalloproteinases (ADAMs) and zincins [139,140]. While these inhibitors decrease the activity of MMPs that would be overexpressed, they also decrease the activity of MMPs that are not related to the pathophysiology of the disease. For this reason, first-generation MMP inhibitors are not the best pharmacological choice in clinical trials. MMP inhibitors based on small molecules that bind to the S1 region near the zinc ion in the catalytic site have been developed, thus allowing more selectivity and specificity to particular substrates [137,138]. Others MMP inhibitors—based on peptides, proteins, antibodies and, more recently, targeted mutations in TIMPs—have increased specificity and selectivity [137,138]. Doxycycline is currently the only FDA-approved MMP inhibitor available in the clinic for periodontites treatment [141,142,143]. It is known as a broad-spectrum MMP inhibitor but preferentially inhibits MMP-2, MMP-9 and MMP-8. It is a weak MMP-1 inhibitor and does not inhibit MMP-3 or MMP-7 [144,145,146,147]. Another inhibitor is Ilmostat (GM6001), which has good specificity for MMP-1, MMP-2 and MMP-9 and has already undergone phase-I and phase-II clinical trials [148]. Prinomastat (AG-3340) has specificity for MMP-1, MMP-2 and MMP-9, as shown in a phase-III clinical study [149]. There is the GA-5745/andecaliximab antibody, with selective activity for MMP-9, which has already undergone phase I, II and III clinical studies [150,151,152]. However, it has not yet been tested in cardiovascular disease trials. With respect to the use of MMP inhibitors, it is suggested that normalizing MMP levels instead of completely inhibiting them is better because there are important physiological roles played by these proteases. Excessive MMP inhibition may lead to bone, joint, inflammatory and cardiovascular complications [61,62,63].

It has not yet been established whether eNOS cofactors such as FAD, FMN and BH4 can interfere with MMP activity. However, supplementation with enzyme cofactors directly increases eNOS enzyme activity and NO production [153,154]. It is possible that increasing these cofactors decreases MMP activity in hypertension because NO can modulate MMP activity [71,72,73,74]. Additionally, a study showed that MMP-2 cleaves heat shock protein 90 (HSP90), another cofactor for eNOS activity, and MMP-2 colocalizes with HSP90 and eNOS, suggesting a direct interaction between MMP-2 and eNOS [155]. Therefore, supplementation with eNOS cofactors could represent an exciting, non-tested therapy to control abnormal MMP activity in hypertension.

In conclusion, it is now clear that imbalanced vascular MMP activity promotes vascular dysfunction and a variety of structural alterations, resulting in vascular remodeling in hypertension. The protective effects of MMP inhibitors, antioxidants and drugs that enhance vascular NO activity have recently been becoming more evident and new therapies are emerging that address these important mechanisms, which may offer significant advantages to prevent the vascular remodeling of hypertensive patients.

## Data Availability

Not applicable.
